# Application of Augmented Reality to Maxillary Resections: A Three-Dimensional Approach to Maxillofacial Oncologic Surgery

**DOI:** 10.3390/jpm12122047

**Published:** 2022-12-12

**Authors:** Francesco Ceccariglia, Laura Cercenelli, Giovanni Badiali, Emanuela Marcelli, Achille Tarsitano

**Affiliations:** 1 Oral and Maxillo-Facial Surgery Unit, IRCCS Azienda Ospedaliero-Universitaria di Bologna, Via Albertoni 15, 40138 Bologna, Italy; 2 Maxillofacial Surgery Unit, Department of Biomedical and Neuromotor Science, University of Bologna, 40138 Bologna, Italy; 3 eDimes Lab-Laboratory of Bioengineering, Department of Experimental, Diagnostic and Specialty Medicine, University of Bologna, 40138 Bologna, Italy

**Keywords:** augmented reality, mixed reality, AR-assisted surgery, CAD-CAM surgery, CAD-CAM cutting guides, HoloLens, head-mounted displays, maxillofacial surgery

## Abstract

In the relevant global context, although virtual reality, augmented reality, and mixed reality have been emerging methodologies for several years, only now have technological and scientific advances made them suitable for revolutionizing clinical care and medical settings through the provision of advanced features and improved healthcare services. Over the past fifteen years, tools and applications using augmented reality (AR) have been designed and tested in the context of various surgical and medical disciplines, including maxillofacial surgery. The purpose of this paper is to show how a marker-less AR guidance system using the Microsoft^®^ HoloLens 2 can be applied in mandible and maxillary demolition surgery to guide maxillary osteotomies. We describe three mandibular and maxillary oncologic resections performed during 2021 using AR support. In these three patients, we applied a marker-less tracking method based on recognition of the patient’s facial profile. The surgeon, using HoloLens 2 smart glasses, could see the virtual surgical planning superimposed on the patient’s anatomy. We showed that performing osteotomies under AR guidance is feasible and viable, as demonstrated by comparison with osteotomies performed using CAD-CAM cutting guides. This technology has advantages and disadvantages. However, further research is needed to improve the stability and robustness of the marker-less tracking method applied to patient face recognition.

## 1. Introduction

In the last fifteen years, tools and applications employing augmented reality (AR) have been designed and tested in the context of several surgical and medical disciplines [1,2,3,4,5,6], including maxillofacial surgery [7,8,9,10]. Dental implantology and orthognathic surgery are the most frequent applications of virtual reality and augmented reality in the oral and cranio-maxillofacial surgery field [9,11,12,13].

AR is a technology that superimposes and displays a virtual three-dimensional model on the operator’s field of view. When using AR as a navigation system, it is possible to provide immediate guidance by intuitively displaying useful information for the surgeon, such as anatomical structure in a deep area or virtual surgical planning (VSP).

The end user may experience the AR view with various display types, such as standard 2D monitors or tablets, projectors [14] or wearable head-mounted displays [15] (HMD).

Nevertheless, as is true of many emerging technologies, no standard method by which AR technology could/should be transferred to clinical practice has yet been developed [14].

Over the past three years, we have employed AR to perform free fibular flap harvesting in a case series of seven consecutive patients undergoing mandibular reconstruction for benign and malignant tumors [15], and to perform galeo-pericranial flap harvesting based on the vasculature of the superficial temporal artery [16].

We also experimentally evaluated the registration accuracy achievable while executing AR-guided maxillofacial surgery tasks, using both commercially available HMD such as HoloLens and “surgery-specific” headsets that are under development [17,18,19].

The aim of this work is to show how a marker-less AR guidance system using the HoloLens 2 Microsoft HMD can be applied in mandible and maxilla demolition surgery.

We describe three mandibular and maxillary oncologic demolitions performed during 2021 using the AR support.

In these three patients, we applied a marker-less tracking method based on recognizing the patient’s face profile. The surgeon using HoloLens 2 smart glasses was able to see the VSP superimposed on the patient’s anatomy.

## 2. Materials and Methods

### 2.1. Clinical Cases

#### 2.1.1. First Patient

**Figure 1:**
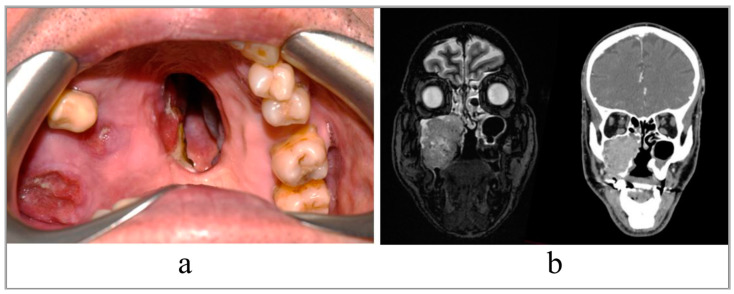
(**a**) Intraoral view of the tumor; (**b**) preoperative RMN and CT scan, coronal slices.

A 68-year-old patient with recurrent squamous cell carcinoma of the right maxilla. The patient was studied with a contrast-enhanced CT scan and MRI of head and neck, which showed an uptake of the dye at the level of a neoformation that infiltrated the right maxillary bone and its sinus, and probably extended to the ipsilateral mandibular branch (Figure 1).

The study of the neck did not reveal any macroscopically significant lymphadenopathy. The patient reported previous surgery, the first 30 years ago and the second about 20 years ago, for removal of clear cell carcinoma of the maxilla and a recurrence. In addition, the patient had suffered an ischemic stroke 13 years earlier, which had left no consequences. 

#### 2.1.2. Second Patient

A 56-year-old patient with osteomyelitis of the left jaw. The patient reported the origin of her symptoms following a molar extraction performed 4 years earlier. During this time, the patient underwent first antibiotic therapy and then surgical curettage. Unfortunately, no treatment gave result. The patient was studied with a contrast-enhanced CT scan which showed the extension of the osteitis, and surgery was planned for mandibular disarticulation resection and reconstruction with custom-made titanium plate and free fibula flap. The day before the surgery, the patient refused reconstruction with the fibula, so it was performed only with the plate.

#### 2.1.3. Third Patient

A 38-year-old patient also suffering from osteomyelitis of the left mandible following an extraction two years earlier. When usual medical treatment failed, the patient was referred to our surgical unit by his dentist. A CT-scan was performed to study the extent of the disease and to plan surgery. Next, the patient underwent mandibular resection and reconstruction with fibula free flap and CAD-CAM titanium plate.

### 2.2. Preparation of AR Guidance Application

**Figure 2:**
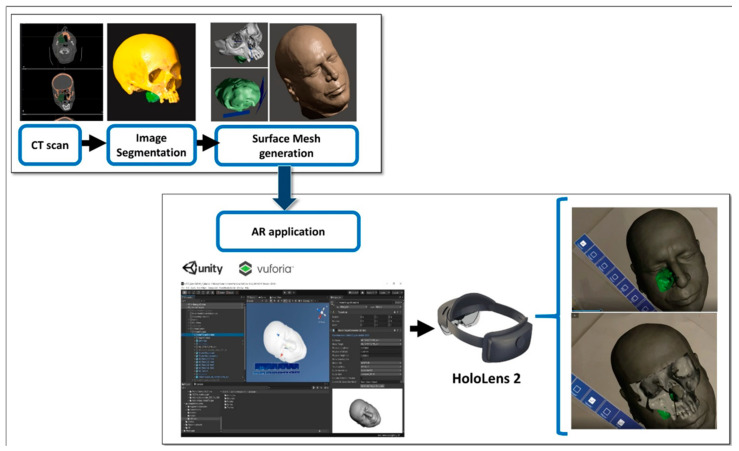
The steps for preparation of the AR guidance application for HoloLens 2 glasses (Redmond, WA, USA).

The steps for preparation of the AR guidance application for HoloLens 2 glasses are depicted in Figure 2. The process started from the acquisition of computed tomography (CT) datasets of the patient. CT scans were acquired with a slice thickness of 0.6 mm (Lightspeed VCT LS Advantage 64 slices; General Electric Medical System).

Anatomical areas of interest for the surgery were segmented using D2P™ software (3D Systems Inc., Rock Hill, SC, USA), a certified software package designed to convert DICOM medical images into 3D digital models. In detail, the maxillary bones, the tumoral mass in the midface region and the facial skin were segmented as separated masks from the CT scan. Then, three-dimensional polygonal surface meshes were generated from each segmented mask and saved in STL (Standard Tessellation Language) format.

**Figure 3:**
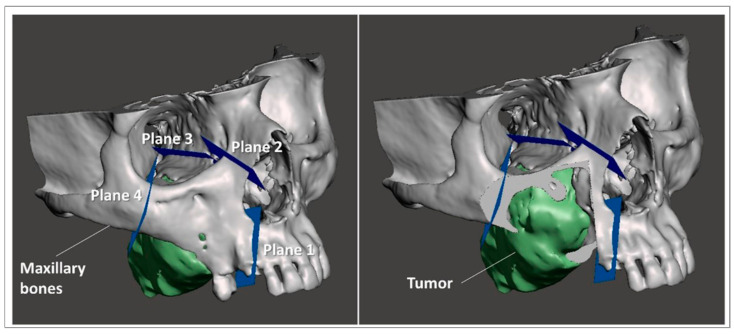
Virtual planes for maxillary bone resections.

Using a 3D modeling software (Meshmixer, Autodesk Inc., San Rafael, CA, USA) virtual planes for maxillary bone resections were planned following the indications of maxillofacial surgeons, and then converted in STL objects (Figure 3).

The obtained virtual models of the anatomical parts and the planned resection planes were imported into Unity 3D (Unity Technologies, San Francisco, CA, USA), a software provided with a specific development kit for creating augmented reality applications (Vuforia Engine package, PTC, Inc., Boston, MA, USA).

The tracking algorithm and the registration between the digital models and the real scene were implemented using the Model Target function of Vuforia Engine, which allows the marker-less tracking of a physical object in the real world by recognition of the shape of the 3D object, itself observed from a certain perspective. A Model Target requires that the user holds the AR display at a particular angle relative to the object, and at a particular distance to initialize the tracking. The application typically draws an image (guide view) showing an approximation of the object’s 3D shape from this distance and viewing angle, so that the user just needs to look at the real object while wearing the AR smart glasses until the object matches this guide view. After that, the tracking of the real object and virtual content overlay can begin.

In this study, the feasibility of AR guidance via HoloLens 2 during maxillary tumor resection was evaluated in two phases of the surgery:

(1). At the beginning of the intervention, before skin incision;
(2). After skin incision, with the surgical field ready for tumor resection.

**Figure 4:**
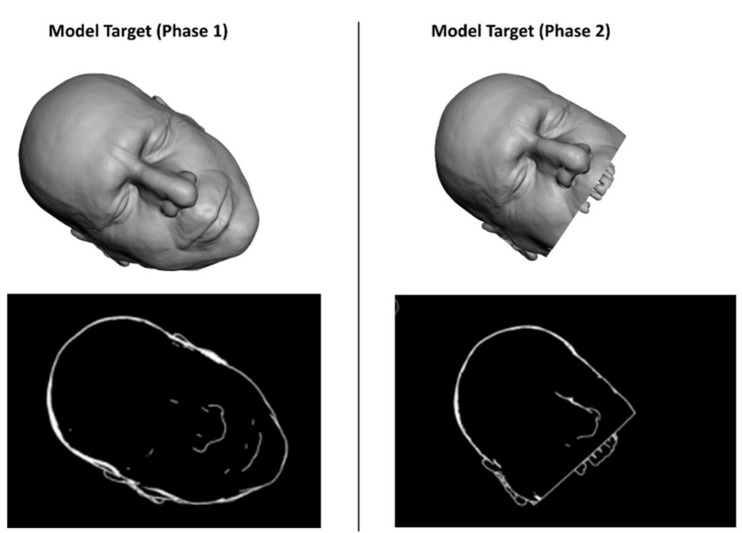
The STL model of the facial skin was used as Model Target for patient tracking.

For each phase, a specific Model Target was generated to optimize the recognition of a 3D shape in the real scene, and the following virtual-to-real scene registration. During phase 1, the STL model of the whole facial skin was used as Model Target for patient tracking, while during phase 2, a portion of the facial skin, trimmed at the level of the mouth and also including the teeth profile, was used (Figure 4).

In both phases, the AR application generates holographic overlays on the real patient, by rendering the underlying anatomical structures (i.e., maxilla and tumor), together with the planned virtual planes for resection.

Interactable user interface toggles (check boxes) were added to turn off and on the rendering of each virtual anatomical structure (facial skin, bones, tumor) and the planned virtual planes. Voice commands to show/hide the virtual structures were also implemented to provide a completely hands-free AR guidance system. (see VIDEO supplementary materials).

A portable high-performance workstation (Intel(R) Core i7-10750H, CPU@ 2.60 GHz, 16 GB RAM, NVIDIA GeForce RTX 2070) was used for the virtual content preparation and for AR development. Then, a UWP (Universal Windows Platform, minimum platform version 10.0.10240.0) app was built to be installed and run onto the HoloLens 2 smart glasses.

The day before surgery, the first surgeon involved in the next day’s intervention was instructed on the use of HoloLens smart glasses and interaction with holograms so that he could feel confident and comfortable with it, and with the developed AR app.

During surgery, the mirroring to an external monitor of what the HoloLens sees was set up in order to share the content view of the smart glasses with other surgeons and operating room assistants.

### 2.3. Preparation of Custom-Made Cutting Guides

Starting from the anatomical 3D reconstruction of the patient’s skull, custom-made cutting guides were designed using 3-Matic software (Materialise NV, Leuven, Belgium), and then 3D printed. In some cases, the cutting guides were manufactured in Polyamide or in Titanium by direct metal laser sintering like the customized reconstructive titanium plate, in other cases in sterilizable Surgical Guide resin (biocompatible Class I) by stereolithography (SLA) 3D printer (Form 3, Formlabs, Somerville, MA, USA).

The cutting guides were used in the operating room to help the surgeon to perform the osteotomies, as well as to evaluate the accuracy of the AR application in providing guidance for the osteotomy lines.

### 2.4. Surgery

The surgical accesses used to approach the tumors were those routinely used in maxillofacial surgery.

**Figure 5:**
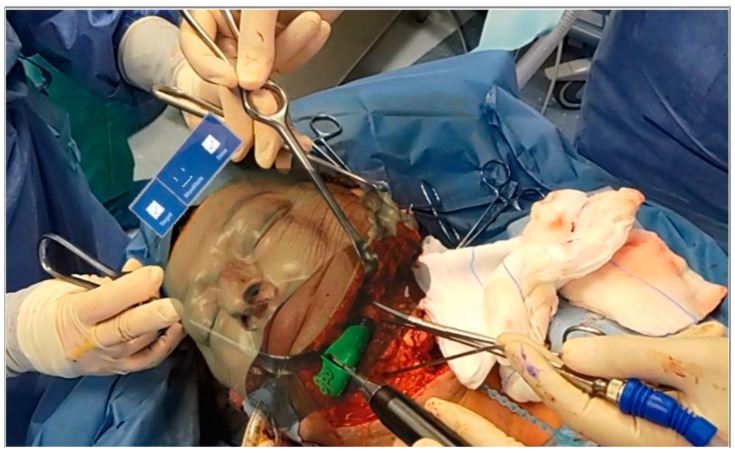
Osteotomies were performed according to the VSP visualized during the surgical act.

Once the maxillaries were skeletonized, osteotomy lines were made using piezoelectric surgery. Osteotomies were performed according to the VSP visualized during the surgical act. In fact, using voice-controlled HoloLens, it was possible to superimpose the surgical planning on the patient’s anatomy. Thus, the surgeon was able to draw the osteotomy lines while simultaneously looking at both the patient and the planning, without the need to look away from the surgical field to look at a navigator monitor (Figure 5).

Once the osteotomy lines were drawn, the custom cutting guides were used for treating the patient. The cutting guides were useful also to evaluate any error in the previously drawn corticotomy line under AR guidance.

Below we detail the surgical procedure of the first patient treated with this methodology.

A Weber–Ferguson surgical incision was performed under general anesthesia. The maxilla was skeletonized to expose the anterior wall of the maxillary sinus and approach the tumor anteriorly. A lip-split mandibulotomy was performed to better approach the tumor posteriorly at the level of the pterygoid laminae.

Before performing the osteotomy lines of the planned type IIIb maxillectomy according to Brown’s classification [20], the surgeon wore HoloLens glasses.

**Figure 6:**
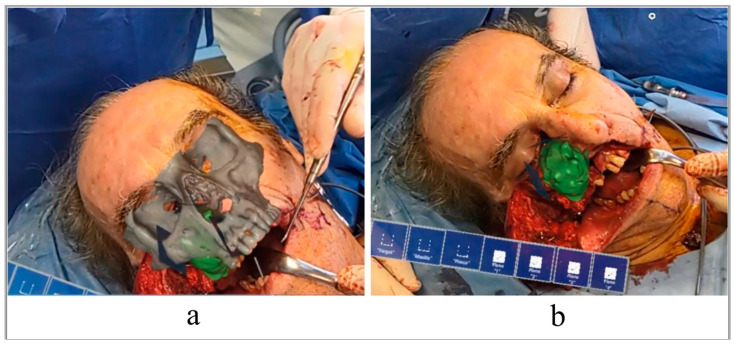
View of the surgical plan superimposed on the patient’s face. With and without virtual bone (**a**,**b**).

The surgeon traced the corticotomy lines by following the VSP superimposed on the patient’s anatomy through the AR glasses. Using voice commands, it was easy for the surgeon to visualize the surgical plan superimposed on the defect. The use of HoloLens HMD allowed a three-dimensional view of the tumor and a better approach to it (Figure 6). Next, the osteotomies were completed following the custom-made cutting guide, and the type IIIb maxillectomy according to Brown was completed.

The corticotomy and osteotomy lines drawn for each spatial plane were compared and measured.

**Figure 7:**
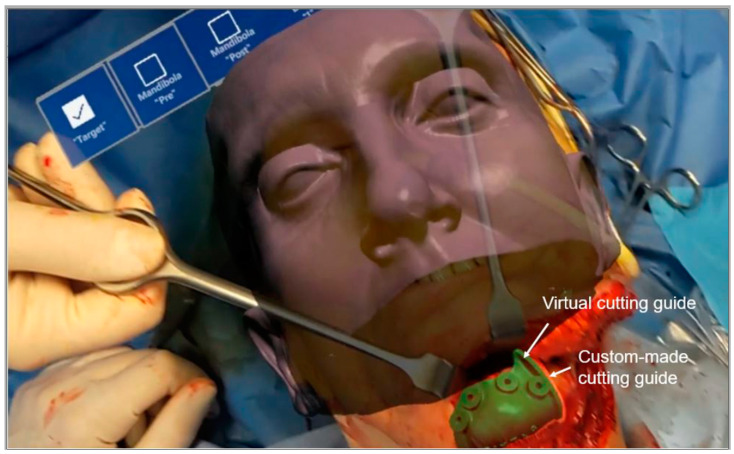
Screenshot of the operative field taken using the HoloLens camera when both the corticotomy line was projected in AR and the cutting guide was positioned on the patient.

To evaluate the distance between the corticotomy line traced using AR and the osteotomy line provided by the custom-made cutting guide, we used a caliper during surgery. Moreover, some screenshots of the operative field were taken using the HoloLens camera when both the corticotomy line was projected in AR and the cutting guide was positioned on the patient. Then, post-operatively, the distance between the two lines was estimated analyzing the recorded intraoperative screenshots (Figure 7).

A functional latero-cervical lymph node emptying was performed according to the guidelines.

Next, the maxillary defect was reconstructed with an antero-lateral thigh flap. The vascular pedicle was anastomosed to the superior thyroid artery and the thyro-lingual trunk. A titanium mesh was used to reconstruct the orbital floor.

## 3. Results

Three patients were treated consecutively with the presented AR guidance approach. They were two males and one female with a mean age of 54 years (56, 38, 68) with recurrent oral squamous carcinoma (SCO) of the right maxilla, and two osteomyelitis of the left mandible.

All surgeries were performed without complications and in line with surgical times for the same pathologies.

There was a total of seven groups of osteotomy planes, each composed of corticotomies performed using AR and the corresponding “real” planes provided by the custom-made cutting guides. Four corticotomy lines and four osteotomy lines were drawn for the first patient, and two for each plane of maxillectomy, representing four groups; One corticotomy and one osteotomy line were drawn for the anterior mandibular resection plane for the second patient, representing one group. Lastly, for the third patient, two corticotomy and two osteotomy lines were drawn, two for each mandibular resection plane, for a total of two groups of osteotomy planes.

For all cases, comparison of the corticotomy lines drawn with the aid of the AR and the osteotomy lines drawn with the use of cutting guides showed a discrepancy under 2 mm.

Following debulking surgery, two patients were simultaneously reconstructed with microvascular-free flaps in accordance with VSP.

## 4. Discussion

Surgery remains the gold standard for the management of maxillofacial neoplasms [21]. CT and MRI represent the most important diagnostic and staging tools for these neoplasms [22]. The combination of these two diagnostic modalities allows a complete evaluation of the tumor in terms of size and relationship with adjacent and deep structures.

An in-depth understanding of the size of the tumor and its relationship with adjacent structures allows resective surgery to be performed with clear margins and without unnecessarily harming anatomical boundaries.

Nowadays, CAD-CAM cutting guides allow the VSP [23] to be brought onto the operating field, even if with many difficulties linked, above all, to their correct positioning. Indeed, in order to position these surgical templates, it is necessary to perform wider surgical accesses with consequent greater damage to the surrounding healthy tissues [24].

The application of intraoperative navigation has represented a valid alternative, effective in terms of cost/benefit balance for the translation of the VSP into operative reality, and with good results in terms of precision and safety [25,26]; however, its broad adoption is limited by higher initial investments and slow technological turnover.

Moreover, current intraoperative navigation systems allow surgeons to inspect and interact with 3D images and objects on a flat-panel display [27], thus limiting anatomical depth perception and forcing a hand-eye coordination mismatch while the surgeon operates on the surgical field and looks at the display simultaneously.

Interferences (e.g., reorientating of the radiographic images, the surgical plan, or equipment issues) during surgical procedures can be deleterious [28].

With holographic imaging technology, it is now possible for users wearing head-mounted displays to manipulate and interact with virtual objects in real time [29].

In this study, HoloLens 2 smart glasses was used as a head-mounted display to project holograms onto the patient’s anatomy. During the various steps of the surgery, the STL model of the whole facial skin was first used as a target model for patient tracking, followed by a portion of the facial skin cut at the level of the mouth and including the profile of the teeth. Throughout the surgery, the AR application generated holographic overlays on the real patient, rendering the jaw and tumor together with the virtual planes planned for resection. This determined several advantages: visualization of the tumor, the critical surrounding structures and the virtual surgical plan intraoperatively, all consistent with the real patient’s anatomy; display of the planned osteotomy line in real time and the ability to follow it without looking away from the surgical table; and manipulation of the augmented digital content using the voice control function supported by the HoloLens HMD which leaves the surgeon’s hands free to execute the surgical task.

In previous studies on mixed reality, the superimposition method was used to overlay holograms over actual anatomical structures, and manual matching was then performed [30,31]. The main drawbacks of manual matching are the time-consuming matching process and the low matching accuracy.

To the best of our knowledge, the application of augmented reality using the patient’s face directly as a model target for patient tracking has not been reported previously in oral and maxillofacial surgery. This allows surgeons to superimpose the holograms of the VSP directly onto the patient’s face without using any marker-based tracking system, thus freeing the surgical field from cumbersome objects that may obstruct visual and manual tasks, or that must always be in the line of sight of an external camera. This provides more surgical room and manual dexterity.

In contrast, a major problem reported in the literature with the use of HoloLens is visual discrepancy. The hologram site may appear in a different spatial position in the assistant’s view, even after registration by the surgeon. Galati et al. reported a discrepancy of 4.5 cm when the same reference point was viewed from different perspectives. Visual discrepancy is potentially dangerous [27].

Another problem that has been reported is that the operating light may affect the quality of the hologram, and that the overlap with the surgical field may obstruct the view of anatomical structures [32]. As previously reported in the literature, the main problem encountered in this study is related to Vuforia’s recognition of the model target. In fact, this marker-less tracking method is sensitive to variations in light and/or color of the object, which does not guarantee optimal performance, especially in terms of time. For this reason, future work should include the use of different technologies for tracking and recognition of the chosen target. In fact, it is well known that during real surgical procedures, there are several obstacles, such as blood or tissues, that can influence the visibility of the patient’s face [33]. In our experience, we have attempted to overcome this problem with the help of voice commands, activating the planning superimposition only when really needed. The results obtained in this work, although recorded on a small sample of patients, resulted in discrepancies of 2 mm between corticotomies drawn using AR, and osteotomies drawn using cutting guides. We believe this difference is a consequence of the registration and limitation of the current tracking method based solely on facial profile recognition. For future studies, we are going to experiment with some other tracking options that could improve registration accuracy, like the use of trackers with QR codes; however, we wish to do this without creating bulk and obstacles in the operating field. A further direction will be to experiment, for these types of tumor surgery, with the use of “surgery-specific” headsets for AR-based intraoperative guidance, e.g., the investigational device VOSTARS (https://www.vostars.eu/, accessed on 6 November 2022), which has already demonstrated on a phantom very promising submillimetric accuracy in guiding high-precision surgical tasks [17,19,34]. Moreover, the HoloLens is relatively bulky and heavy, and wearing it for long periods can be uncomfortable. Despite this, a considerable advantage is that when using Microsoft^®^ HoloLens 2, it is possible to look at the real environment around you, as it is an “optical see-through (OST)” head-mounted display (HMD); i.e., it uses semi-transparent surfaces of projection that preserve the user’s direct view of the world. This aspect confers a clear advantage, particularly when used to interact with objects in the peripersonal space, as it allows the user to maintain an unaltered and almost natural visual experience of the surrounding world.

## 5. Conclusions

The aim of this study was to evaluate the feasibility of performing a resective maxillary osteotomies using augmented reality.

The use of augmented reality appears to be safe and effective for tumor resection in the oral and maxillofacial region. This technology has advantages and disadvantages. However, further research that aims to improve the stability and robustness of the marker-less tracking method applied to patient face recognition is needed.

## Data Availability

Not applicable.

## Supplementary Materials

The following supporting information can be downloaded at: https://www.mdpi.com/article/10.3390/jpm12122047/s1, Video S1: Movie showing the AR application in an oncologic maxillectomy
